# The emergence of functional connectivity patterns bound by an underlying structural connectivity substrate

**DOI:** 10.1186/1471-2202-12-S1-P300

**Published:** 2011-07-18

**Authors:** Helen Saad, Gabriel Silva

**Affiliations:** 1 Department of Bioengineering, University of California San Diego, La Jolla, CA 92093, USA; 2 Department of Ophthalmology, University of California San Diego, La Jolla, CA 92037-0946, USA; 3 Neurosciences Graduate Program, University of California San Diego, La Jolla, CA 92093, USA

## 

A major challenge in neuroscience is to determine the rules that govern the structural and functional interdependence of cellular neural circuits and networks in the brain [1]. This challenge is a difficult one given the fact that brain networks are complex networks with emergent behaviors and properties. Understanding the underlying mechanisms that shape the intimate relationship between structure and function will help us better understand how functional brain states emerge from their underlying structural substrate, in addition to providing us with new insights into how the disruption of this function-structure interdependence will affect brain function.

It is clear that structure and function are interrelated and that specific brain regions and even specific brain networks have well defined structural topologies, determined by the very specific neuron types that they recruit and the specific connections that these neurons project [2]. This fundamental property plays a major role in determining functions that are characteristic of certain brain networks. However, it is less clear how experience and exposure to external cues can lead to the emergence of fast and flexibly reconfigured functional networks that are bound by a well defined structural substrate, and how this structural substrate in turn, but at a slower time scale, may reshape functional networks. Even more intriguing is the fact that such functional networks, while highly dynamic, play a major role in remodeling, associating and storing information that is presented by external cues for time periods that can sometimes extend over an individual’s lifetime; in short they underlie learning and memory.

Despite considerable advances in experimental techniques and computational paradigms, our understanding of this problem is still incomplete. We have begun to address this issue by developing a model that is composed of integrate-and-fire excitatory and inhibitory neurons that are connected to external inputs and project recurrent connections among themselves. It is based on the hypothesis that functional network dynamics are bound by three main rules: (1) Structural network connectivity is predetermined to a high extent and is a prerequisite for functional connectivity to take place between two neurons. (2) Synaptic plasticity that the network undergoes is Hebbian-like and weight change (following potentiation or depression) is proportional to the number of afferents of a certain neuron. (3) Heterosynaptic plasticity takes place to help regulate the neuronal firing rate. Our results show that structure and synaptic plasticity determine function at large and sets boundaries to the information capacity of the neuronal network. We also tested different network sizes and different physical connectivity constraints in order to determine how the emergent functional dynamics and network capacity scale with the network size. Here we present and discuss our data and results to date with this model.

We aim, through this theoretical study and in the long run, to come full circle to a better and more integrated understanding of the intricate interdependence between structure and function in brain networks as shaped by learning and memory. Unraveling these dynamics will directly contribute to our understanding of how different parts of the mammalian brain function in health, how they fail in disease, and how best to guide nervous system related therapies.
